# Vascular-targeted TNFα and IFNγ inhibits orthotopic colorectal tumor growth

**DOI:** 10.1186/s12967-016-0944-3

**Published:** 2016-06-24

**Authors:** Jing Shen, Zhi Jie Li, Long Fei Li, Lan Lu, Zhan Gang Xiao, William Ka Kei Wu, Lin Zhang, Ming Xing Li, Wei Hu, Kam Ming Chan, Chi Hin Cho

**Affiliations:** Laboratory for Molecular Pharmacology, Department of Pharmacology, School of Pharmacy, Southwest Medical University, Luzhou, People’s Republic of China; School of Biomedical Sciences, Faculty of Medicine, The Chinese University of Hong Kong, Shatin, NT, Hong Kong, People’s Republic of China; Department of Anaesthesia and Intensive Care, The Chinese University of Hong Kong, Hong Kong, People’s Republic of China; Harry Perkins Institute of Medical Research, University of Western Australia, Crawley, WA 6009 Australia

**Keywords:** Vascular targeting, TNFα, IFNγ, Colorectal cancer, Drug delivery

## Abstract

**Background:**

Tumor necrosis factor alpha (TNFα) and interferon gamma (IFNγ) were originally identified to show potent anti-tumor activity and immunomodulatory capability. Unfortunately, several clinical studies of relevant cancer therapy did not observe significant response in maximum tolerated dose whether given alone or in combination. We have identified a tumor vasculature homing peptide (TCP-1 peptide) which targets only the vasculature of colorectal tumors but not normal blood vessels in animals and humans. In the current study, the antitumor effect of TCP-1/TNFα and TCP-1/IFNγ alone or in combination was studied in orthotopic colorectal tumor model.

**Methods:**

TCP-1/TNFα and TCP-1/IFNγ recombinant proteins were prepared and i.v. injected to study the in vivo anticancer effect in orthotopic colorectal tumor model. Tumor apoptosis was determined by TUNEL staining and cleaved caspase-3 immunofluorescent staining. Tumor infiltrating lymphocytes were analyzed by immunofluorescent staining and flow cytometry. Western-blot was performed to examine the expression of proteins. Cell apoptosis was measured by Annexin V/PI flow cytometry.

**Results:**

Targeted delivery of TNFα or IFNγ by TCP-1 peptide exhibited better antitumor activity than unconjugated format by inducing more tumor apoptosis and also enhancing antitumor immunity shown by increased infiltration of T lymphocytes inside the tumor. More importantly, combination therapy of TCP-1/TNFα and TCP-1/IFNγ synergistically suppressed tumor growth and alleviated systematic toxicity associated with untargeted therapy. This combination therapy induced massive apoptosis/secondary necrosis in the tumor.

**Conclusions:**

Taken together, our data demonstrate TCP-1 is an efficient drug carrier for targeted therapy of colorectal cancer (CRC). TCP-1/TNFα combined with TCP-1/IFNγ is a promising combination therapy for CRC.

**Electronic supplementary material:**

The online version of this article (doi:10.1186/s12967-016-0944-3) contains supplementary material, which is available to authorized users.

## Background

Despite a slight decline in the mortality rate over the past decade, colorectal cancer (CRC) remains the third most common cancer and a leading cause of cancer deaths worldwide. Tumor necrosis factor alpha (TNFα) consists of three noncovalently linked TNFα monomers, ~17.5 kDa each, which forms a compact bell-shaped homotrimer [1, 2]. TNFα is a powerful anti-tumor cytokine as well as a potent inflammatory cytokine which can induce complex immune responses [3]. It exerts its anti-tumor activity through complex mechanisms including induction of inflammatory and immune responses, tumor cell apoptosis/necrosis and extensive thrombosis and destruction of tumor vasculature [4, 5]. Although TNFα shows potent anti-tumor activity and produces impressive results in various animal cancer models, clinical use of TNFα as an anticancer drug is hampered by severe systemic toxicity [6, 7]. To date, the clinical use of TNFα has been limited to cancer treatment in the isolated limb perfusion (ILP) setting for soft tissue sarcoma and melanoma intrinsic metastases confined to the limb [8, 9].

It has been shown that tumor vascular-targeted delivery of TNFα is capable of increasing tumor concentration of TNFα and directing TNFα specifically to the tumor site [6, 10–12]. This strategy has resulted in several tumor vascular ligands fused to TNFα for cancer therapy research, and even clinical trials are underway [13–16]. We have previously established an orthotopic colorectal tumor model and identified a cyclic peptide known as TCP-1. This peptide can specifically target the vasculature of orthotopic colorectal tumors [17]. Targeted delivery of TNFα by TCP-1 peptide displayed more potent antitumor activity than unconjugated TNFα by inducing more apoptosis and destructing neovasculature in orthotopic colorectal tumors at 24 h with the dose 5 μg/mouse. Furthermore, low-dose TCP-1/TNFα (1 ng/mouse) potentiated the antitumor effect of 5-fluorouracil (5-FU) by normalizing the tumor vasculature, facilitating the infiltration of immune cells to the tumor as well as improving 5-FU penetration into the tumor mass. More importantly, TCP-1/TNFα attenuated the immunosuppressing effects of TNFα in bone marrow and spleen with marked reduction in systemic toxicity [18]. These findings provide a solid proof that TCP-1/TNFα could be used to treat CRC through synergistic effects with standard chemotherapeutic agents as targeted therapy.

Interferon gamma (IFNγ) is a pleiotropic cytokine produced by immune cells and plays physiologically important roles in promoting innate and adaptive immune responses [18, 19]. It is also indicated that IFNγ could induce the antiproliferative and proapoptotic effects on various tumor cells, repress tumor angiogenesis and produce antitumor cellular responses through activation of natural killer cells, macrophages and CD8^+^ cytotoxic T cells [20]. But similar to TNFα, IFNγ lacks a unique selectivity as an antitumor agent and can bind to most of cells due to ubiquitous expression of its receptor [21]. Therefore, IFNγ failed in several clinical trials as a sole antitumor agent due to low maximal tolerated dose in patients [19, 21]. Tumor vasculature targeting approach also has been used to deliver IFNγ to tumor tissues for targeted therapy. Several studies of targeted IFNγ have obtained promising results [21–23]. Low dose of targeted IFNγ can produce significant tumor growth inhibition in different tumor types [22].

TNFα and IFNγ have been shown to have synergistic antitumor effect in a few cell line studies [24–27]. Relevant study also demonstrated that co-administration of targeted TNFα and nontargeted IFNγ resulted in significant synergistic tumoricidal activity in renal cell carcinoma. However, early clinical studies failed in patients with advanced gastrointestinal cancers through co-administration of non-targeted TNFα and IFNγ [28–31]. Targeted delivery of TNFα or IFNγ not only reduces the effective dose of individual cytokines and enhances their local concentration in tumors, but also enlarges their therapeutic window. More importantly it is likely that the combination of targeted TNFα and IFNγ might produce more effective antitumor activity than either agent given alone. This could further reduce the dosage of each agent, which will markedly reduce the potential side effects produced by these agents and make the treatment more viable in cancer patients. Here, we explored the antitumor effect of targeted TNFα or IFNγ in CRC through using fusion protein of TNFα or IFNγ with TCP-1 peptide, a novel ligand which can specifically bind to the vasculature of orthotopic colorectal tumors [24–27]. Furthermore, the antitumor activity and mechanism of action for each fusion protein alone or in combination were also assessed.

## Methods

### Cells and animals

The murine CRC cell Colon 26 was obtained from the Health Science Research Resources Bank (Osaka, Japan). Mouse fibroblast cell line L929 for IFNγ activity determination was purchased from the Shanghai Cell Bank of the Chinese Academy of Sciences (Shanghai, China). Cells were grown in RPMI 1640 supplemented with 100 U/mL penicillin G, 100 μg/mL streptomycin, and 10 % fetal bovine serum (FBS) and maintained at 37 °C in a humidified atmosphere containing 5 % CO_2_. Male BALB/c mice aged 6 weeks were maintained at the Chinese University of Hong Kong Animal Facility. Animal experiments in this project had been approved by the Laboratory Animals Ethics Committee of the Chinese University of Hong Kong.

### Reagents and antibodies

Anti-mouse CD31 (553274), anti-mouse CD4 (553043) and anti-mouse CD8 (550281) monoclonal antibodies were purchased from BD Pharmingen. Anti-caspase-3 (9662), anti-PARP (9542), anti-β-actin (4967) antibodies were obtained from Cell Signaling Technology. PE-Cy™ 7 anti-mouse CD3 (100320), PE anti-mouse CD4 (103405) and FITC anti-mouse CD8a (100706), APC anti-mouse CD34 (128612), PE-Cy™ 7 anti-mouse CD45 (103222) antibodies, and isotype IgG were purchased from Biolegend. In Situ Cell Death Detection Kit (Fluorescein) was brought from Roche. Dead Cell Apoptosis Kit with Annexin V FITC and PI was purchased from ThermoFisher.

### Orthotopic CRC model

Animals were anesthetized with a mixture of ketamine and xylazine. A 29-gauge syringe was used to inject 2.5 × 10^4^ colon 26 cells, suspended in RPMI 1640 with 10 % FBS, submucosally into the distal, posterior rectum. The injection was performed approximately 1–2 mm beyond the anal canal and into the rectal mucosa, which minimized the chance of establishing anal tumors. Tumors were developed at 1.5–2 weeks. Successful models were used for various in vivo anti-cancer experiments after tumors were formed.

### Plasmid construction

The IFNγ and TCP-1/IFNγ plasmids were constructed using method similar to that previously described for TNFα and TCP-1/TNFα [18]. The mouse IFNγ fragment was amplified by PCR from mouse spleen cDNA with primer pair 5′-CAT GGT ACC CAC GGC ACA GTC ATT GAA AGC CTA-3′and 5′-CAT GGA TCC TCA GCA GCG ACT CCT TTT CCG CTT C-3′ flanked by KpnI and BamHI restriction enzyme sites. The PCR product was then cloned into a modified pET-14b vector. Subsequently, the TCP-1 gene was introduced into constructed pET-14b/IFNγ plasmid by PCR-mediated site-directed mutagenesis with the primer pair 5′-TTT TCG CAT TGC GGA GGT ACC CAC GGC ACA GTC ATT GAA AGC CTA-3′ and 5′-AGG ACT AGG CGT ACA AGC GGG CCC CAT ATG GCT GCC GCG CGG-3′. All the constructs were finally confirmed by DNA sequencing. The flow chart of plasmid construction is shown in Additional file 1: Fig. S1.

### Protein expression, purification and verification

The protein expression and purification procedure was performed as previously described [18]. Proteins with >90 % purity based on SDS-PAGE image were used for various examinations. The quantitative chromogenic Limulus amebocyte lysate (LAL) test was used to quantitate Gram-negative bacterial endotoxin. The endotoxin concentration in the purified proteins used in the study is approximately 0.1 EU/μg.

### IFNγ activity assay

The activities of IFNγ and TCP-1/IFNγ were determined in L929 and Colon 26 cells by MTT assay. Briefly, 2 × 10^3^ cells/well were seeded in a 96-well plate with 100 μL growth medium. After 24 h, different concentrations of IFNγ and TCP-1/IFNγ were added for an additional 72 h. Subsequently, 3-(4,5-dimethylthiazol-2-yl)-2,5-diphenyltetrazolium bromide (MTT) was used to detect the cell viability.

### Animal treatment

To investigate whether TCP-1 peptide is able to deliver IFNγ to tumor blood vessels, 50 nmol TCP-1/IFNγ fusion protein or equal molar IFNγ was i.v. injected through tail vein into mice bearing orthotopic CRC to examine the distribution of IFNγ. The proteins were allowed to circulate for 1 h. Tumor and control tissues were collected and prepared for frozen section. Blood vessels were stained with anti-CD31 antibody. IFNγ signal was amplified by biotin-labeled anti-His tag antibody. To study the short-term effect of IFNγ or TCP-1/IFNγ, 5 μg of IFNγ or equal dose of IFNγ in TCP-1/IFNγ, was i.v. injected into mice bearing orthotopic CRC. Animals were sacrificed 24 h after injection. For antitumor experiment, mice bearing orthotopic CRC were randomized into different group (n ≥ 4): PBS, TNFα (1 μg/mouse), TCP-1/TNFα (1 μg TNFα/mouse), IFNγ (5 μg/mouse), TCP-1/IFNγ (5 μg IFNγ/mouse), TNFα (0.5 μg/mouse) plus IFNγ (2.5 μg/mouse) and TCP-1/TNFα (0.5 TNFα μg/mouse) plus TCP-1/IFNγ (2.5 μg IFNγ/mouse). Treatment was given by i.v. injection again through tail vein once. Mice were euthanized at 7 days after drug administration. Tumors and control organs were dissected and prepared for frozen sections. Tumor microvessel density and apoptosis were assessed.

### Histology

At the end of experiment, mice were heart-perfused with 4 % neutral-buffered paraformaldehyde and tumors were obtained for frozen section. Frozen sections were processed and immunofluorescent and immunohistochemical staining were performed as previously described [18].

### Western blots and flow cytometry

Western blots and flow cytometry were performed following the standard laboratory protocol as previously reported [18].

### Hochest and PI double staining

Frozen tissue sections were washed in PBS and double-stained with propidium iodide (PI, 2.5 mg/mL) and Hoechst 33342 (2.5 mg/mL) for 10 min. Intact blue nuclei, condensed/fragmented blue nuclei, condensed/fragmented pink nuclei, and intact pink nuclei were considered viable, early apoptotic, late apoptotic and necrotic cells, respectively.

### Statistical analysis

The results are expressed as mean ± SEM. GraphPad Prism 5 (GraphPad Software) was used for statistical analysis. Two tailed Student’s t test was applied for paired data analysis. For the in vivo treatment experiment with TCP-1 fusion proteins, comparisons among all groups were analyzed by one-way ANOVA followed by the Tukey’s test. P value below 0.05 was considered statistically significant.

## Results

### Targeted delivery of IFNγ by TCP-1 in the orthotopic CRC model

**Fig. 1:**
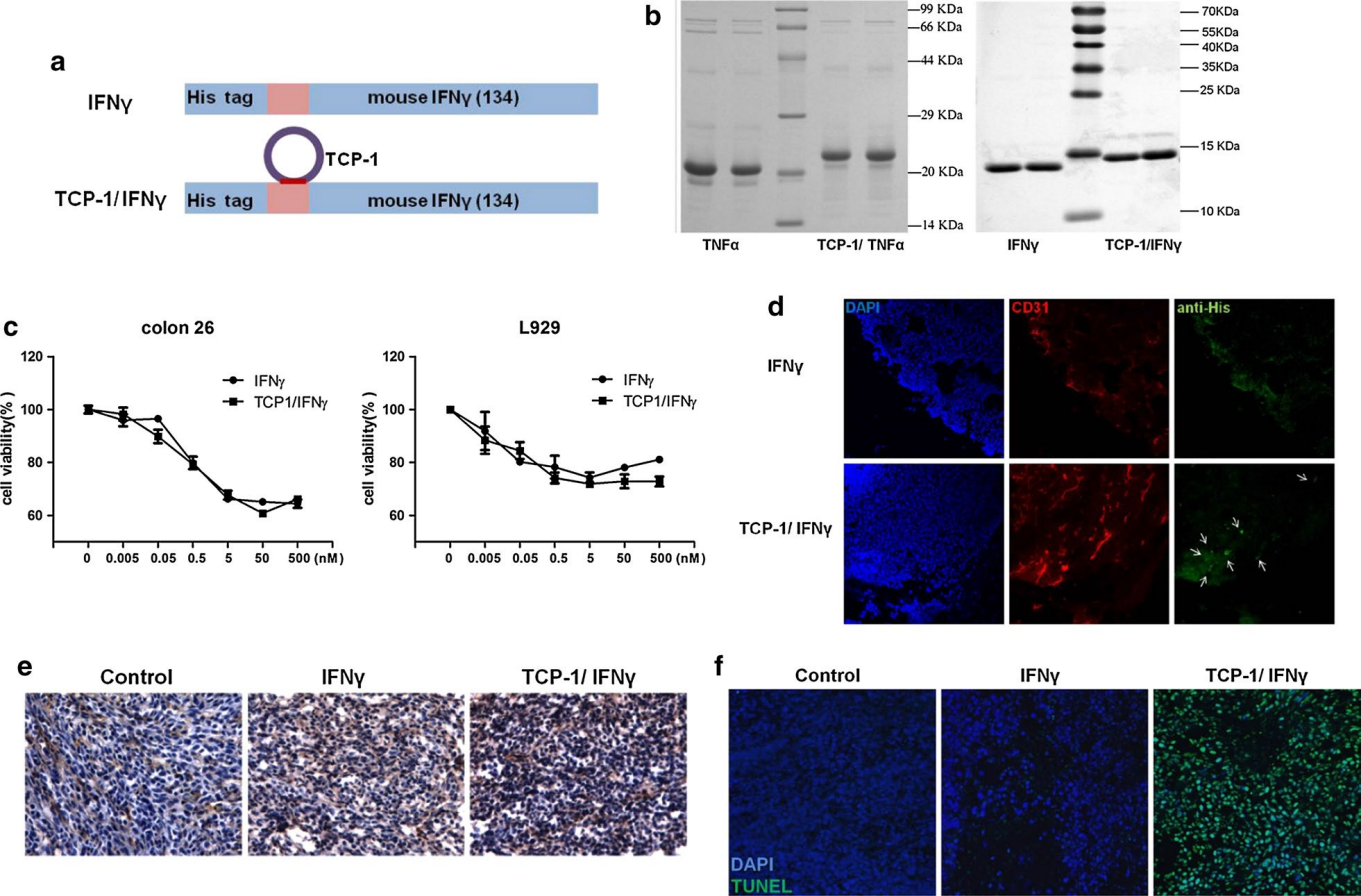
Purification, targeted delivery and functional characterization of IFNγ and TCP-1/IFNγ. **a** Schematic representation of TCP-1/IFNγ and IFNγ fusion proteins. The TCP-1 peptide was fused to N-terminal of IFNγ protein. **b** Purification of TNFα, TCP-1/TNFα, IFNγ and TCP-1/IFNγ. Recombinant proteins were purified using Ni–NTA resin followed by SDS-PAGE and coomassie blue staining. **c** Activity analysis of IFNγ and TCP-1/IFNγ on L929 and Colon 26 cells. Cell viability was determined by MTT assay. **d** 50 nmol IFNγ or TCP-1/IFNγ was i.v. injected into tumor-bearing mice. Mice were sacrificed 1 h later and localization of IFNγ or TCP-1/IFNγ was detected by anti-His tag antibody (*green*). *White arrows* indicate areas where TCP-1/IFNγ was colocalized with CD31 (*red*) in the tumor blood vessels. IFNγ alone did not bind to the blood vessels of tumor tissues. **e** Immunohistochemistry of MHC-I (H-2K^b^). 5 μg IFNγ or TCP-1/IFNγ was i.v. injected into tumor-bearing mice. Mice were sacrificed 24 h later. **f** 5 μg IFNγ or TCP-1/IFNγ was i.v. injected into tumor-bearing mice. Mice were sacrificed 1 h later. Apoptotic cells (*green*) in tumor mass were detected by TUNEL assay. TCP-1/IFNγ could obviously induce more apoptosis of tumor cells when compared with the non-targeted IFNγ

IFNγ and TCP-1/IFNγ plasmids were constructed by similar procedures with that of TNFα and TCP-1/TNFα (Fig. 1a; Additional file 1: Fig. S1). The respective proteins were expressed in *E. coli* and purified by Ni–NTA. Reducing SDS-PAGE of TNFα and TCP-1/TNFα mainly showed a single band of around 20 kD (TNFα, 20 kD; TCP-1/TNFα, 22 kD). Consistent with the fact that IFNγ could form a homodimer, reducing SDS-PAGE of IFNγ and TCP-1/IFNγ mainly showed a single band of around 13 kD (IFNγ, 13 kD; TCP-1/IFNγ, 15 kD), which was expected for monomeric IFNγ (Fig. 1b), whereas nonreducing SDS-PAGE of these two proteins showed two bands of around 13, 26 kD, corresponding to monomers and dimmers respectively (data not shown). The cytostatic activities of IFNγ and TCP-1/IFNγ were determined in a murine fibrosarcoma cell line (L929) and a mouse colonic adenocarcinoma cell line (Colon 26). The effect of TCP-1/IFNγ had no obvious difference from that of IFNγ as determined by the standard cytotoxicity assay (Fig. 1c). Fusion of IFNγ with TCP-1 did not affect the cytotoxicity of IFNγ on these cell lines. These findings indicated that TCP-1 peptide did not change IFNγ folding, oligomerization, activity and binding to IFNγ receptors, thereby producing equipotent cytotoxicity on cells. To directly test whether TCP-1 peptide could deliver IFNγ to tumor vasculature, we injected 50 nmol IFNγ or TCP-1/IFNγ into mice bearing orthotopic CRC through tail veins. Data showed that TCP-1/IFNγ could colocalize with tumor vasculature (Fig. 1d), but not with blood vessels in normal organs including brain, heart and normal colon tissues (Additional file 2: Fig. S2), indicating that IFNγ protein did not negatively affect the binding ability of TCP-1 to tumor vasculature. It has been well reported that IFNγ could up-regulate MHC-I expression [32]. Therefore, the effect of IFNγ and TCP-1/IFNγ on MHC-I expression was determined in vivo. After 24 h treatment, TCP-1/IFNγ induced more MHC-I expression inside the tumor than control or IFNγ (Fig. 1e), indicating more IFNγ was accumulated in the tumor by targeted delivery by TCP-1 peptide. We next briefly investigated the therapeutic effect of TCP-1/IFNγ in vivo. Results showed that TCP-1/IFNγ given 24 h induced more apoptosis when compared to the control and IFNγ groups (Fig. 1f).

### TCP-1/TNFα or TCP-1/IFNγ given alone inhibited orthotopic colorectal tumor growth

**Fig. 2:**
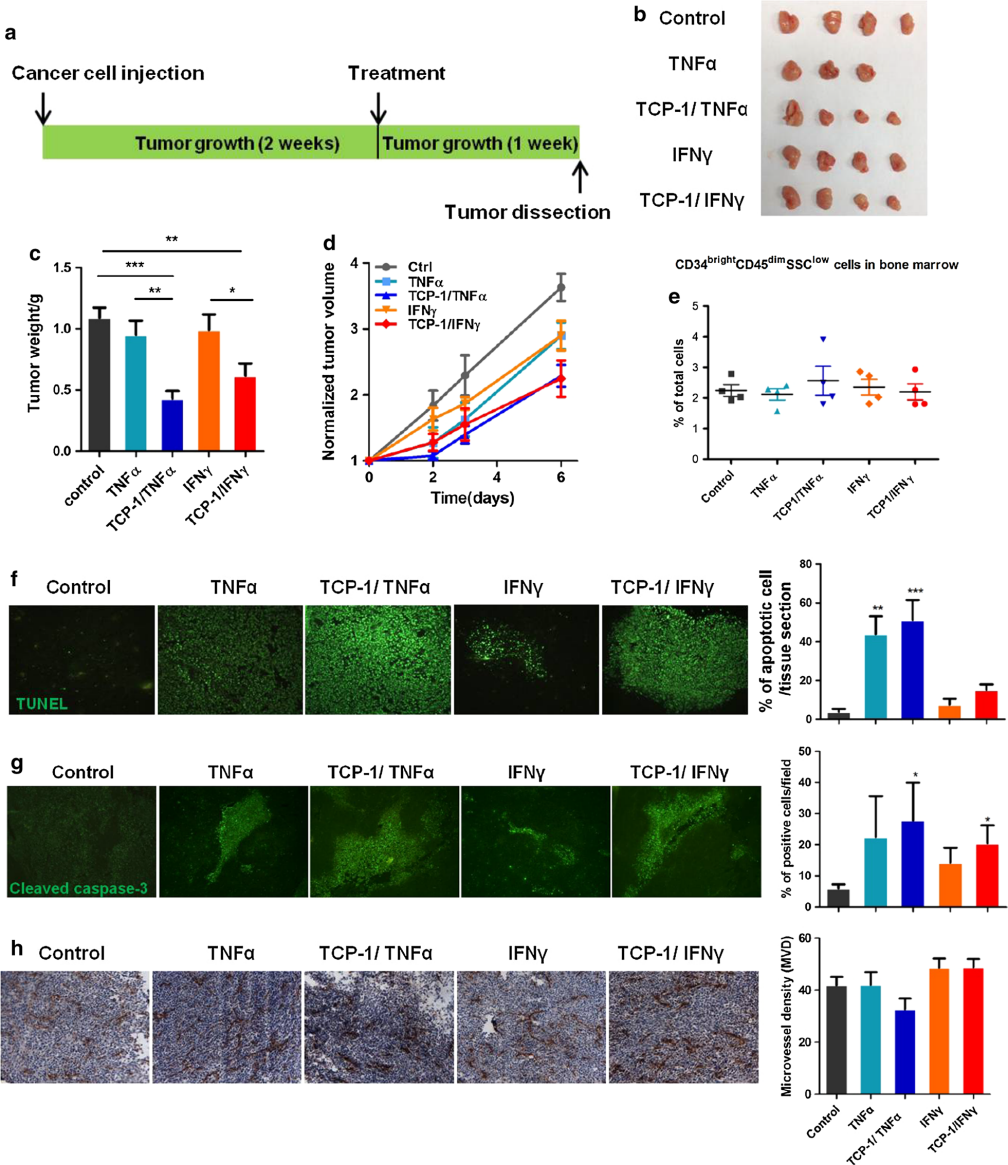
Antitumor activity of TNFα, TCP-1/TNFα, IFNγ or TCP-1/IFNγ single treatment in the orthotopic CRC model. **a** Schematic representation of treatment regimen in the orthotopic CRC model. **b** Representative picture of the tumors in all groups after treatment for 7 days (n ≥ 4 per group). **c** Tumor weight at sacrifice and **d** Tumor size along with time in all treatment groups. Tumor size of each mouse was normalized to that measured at beginning of treatment. TCP-1/TNFα and TCP-1/IFNγ significantly inhibited tumor growth compared with the control group and their respective unconjugated proteins. **e** CD34^bright^ CD45^dim^ hematopoietic cells in bone marrow. There was no myelosuppression in all treatment groups. **f** Apoptosis in tumor mass was detected by TUNEL assay and quantified. There were more TUNEL positive cells in the TNFα and TCP-1/TNFα treated groups compared with the control group. **g** Immunofluorescence staining for cleaved caspase-3 and quantification result. Compared with the control group, TNFα and IFNγ groups showed a little but not significant increase in cleaved caspase-3 while TCP-1/TNFα and TCP-1/IFNγ groups significantly increased cleaved caspase-3. **h** Immunohistochemistry staining for CD31 showing the effect of treatment on tumor blood vessel. There was no obvious vessel destruction for all treatment groups. Data were presented as mean ± SEM. *P < 0.05. **P < 0.01. ***P < 0.001

We next determined the in vivo antitumor activity of TCP-1/TNFα and TCP-1/IFNγ (Fig. 2a). We used the dose of 1 μg/mouse for TNFα and TCP-1/TNFα and 5 μg/mouse for IFNγ and TCP-1/IFNγ according to previous experiment which indicated that 5 μg TCP-1/IFNγ can induce maximal anti-tumor effect (Additional file 3: Fig. S3) [18]. Compared with the control group, TNFα or IFNγ slightly but not significantly suppressed tumor growth, while TCP-1/TNFα or TCP-1/IFNγ significantly decreased tumor size compared with their unconjugated counterparts (Fig. 2b–d). This suggests that conjugation with TCP-1 peptide magnified the antitumor activity of TNFα and IFNγ. For all proteins, there were no myelosuppression observed at indicated dosage as reflected by similar levels of CD34^bright^ CD45^dim^ hematopoietic cells in the bone marrow (Fig. 2e). To further elucidate the antitumor action of TCP-1/TNFα and TCP-1/IFNγ, the level of apoptosis was examined. Concordantly, both TUNEL staining (Fig. 2f) and immunofluorescence staining for cleaved caspase-3 (Fig. 2g) revealed that TNFα or IFNγ increased the number of apoptotic cells inside the tumor while conjugation with TCP-1 further induced apoptosis. Proteins given at the indicated doses did not cause significant vessel destruction (Fig. 2h).

### TCP-1/TNFα combined with TCP-1/IFNγ dramatically inhibited orthotopic colorectal tumor growth

**Fig. 3:**
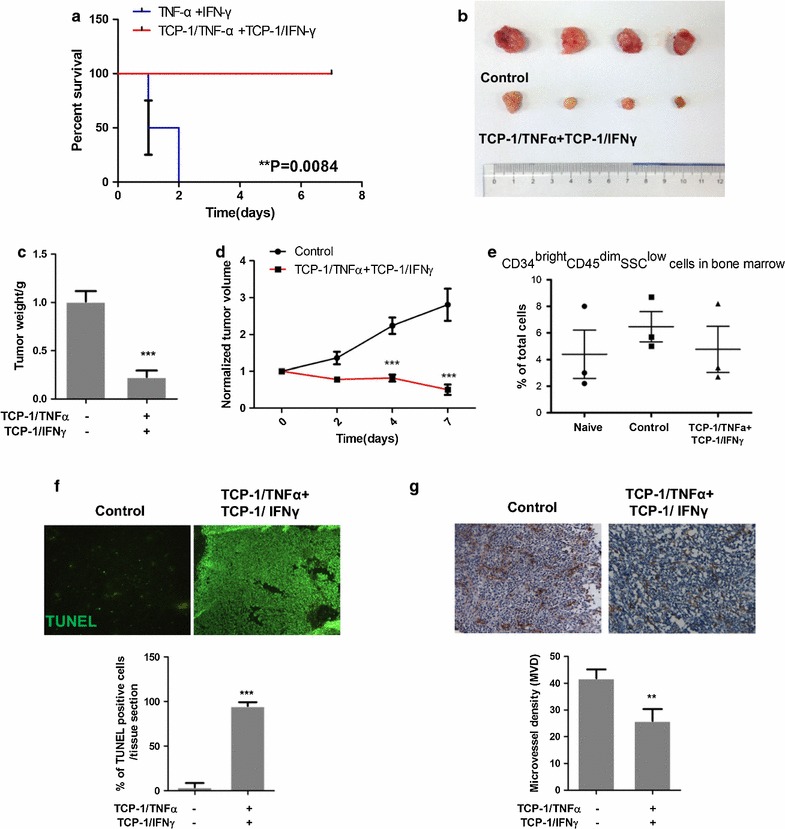
Antitumor activity of TCP-1/TNFα in combination with TCP-1/IFNγ in the orthotopic CRC model. **a** Survival comparison between nontargeted and targeted TNFα and IFNγ combined treatment groups. TCP-1/TNFα and TCP-1/IFNγ combination group significantly improved survival compared with the nontargeted TNFα and IFNγ group. **b** Representative picture of the tumors in control and combined groups after treatment for 7 days (n ≥ 4 per group). TCP-1/TNFα and TCP-1/IFNγ combination treatment dramatically inhibited tumor growth. **c** Tumor weight at sacrifice and **d** Tumor size along with time in all treatment groups (n ≥ 4 per group). **e** CD34^bright^ CD45^dim^ hematopoietic cells in bone marrow. No myelosuppression was observed for TCP-1/TNFα in combination with TCP-1/IFNγ. **f** Representative picture of TUNEL staining and quantification. Combination treatment increased TUNEL positive cells inside the tumor. **g** Representative picture of CD31 immunohistochemistry staining and quantification. TCP-1/TNFα in combination with TCP-1/IFNγ decreased tumor blood vessel. Data were presented as mean ± SEM. **P < 0.01. ***P < 0.001

To determine the combined effect of TCP-1/TNFα and TCP-1/IFNγ on tumor growth, mice were divided into three groups: control (PBS), TNFα (0.5 μg/mouse) combined with IFNγ (2.5 μg/mouse) and TCP-1/TNFα (0.5 μg/mouse) combined with TCP-1/IFNγ (2.5 μg/mouse). Due to systemic toxicity, mice given TNFα combined with IFNγ all died within 2 days while the group given the same dose but conjugated with TCP-1 all survived (Fig. 3a). The data demonstrated that targeted delivery of TNFα combined with IFNγ by TCP-1 peptide drastically inhibited tumor growth than single treatment (Fig. 3b–d; Additional file 4: Fig. S4A, B) and at the same time alleviated systematic toxicity induced by non-targeted TNFα and IFNγ. More importantly, TCP-1/TNFα combined with TCP-1/IFNγ did not reduce CD34^bright^ CD45^dim^ hematopoietic cells in the bone marrow (Fig. 3e), suggesting no myelosuppression was caused. Although there was a little body weight drop at 2 days after treatment (Additional file 4: Fig. S4C), it gradually caught up and there was no obvious toxicity observed in normal organs including colon, liver, kidney, etc. (Additional file 4: Fig. S4D). TCP-1/TNFα combined with TCP-1/IFNγ induced massive cell death in the tumor (>90 %) as shown by the TUNEL staining (Fig. 3f) and also cell death of the tumor vasculature (Fig. 3g). Our results indicated that targeted delivery of these two cytokines by TCP-1 peptide holds great promise for colorectal cancer therapy.

### TCP-1/TNFα and TCP-1/IFNγ enhanced antitumor immunity

**Fig. 4:**
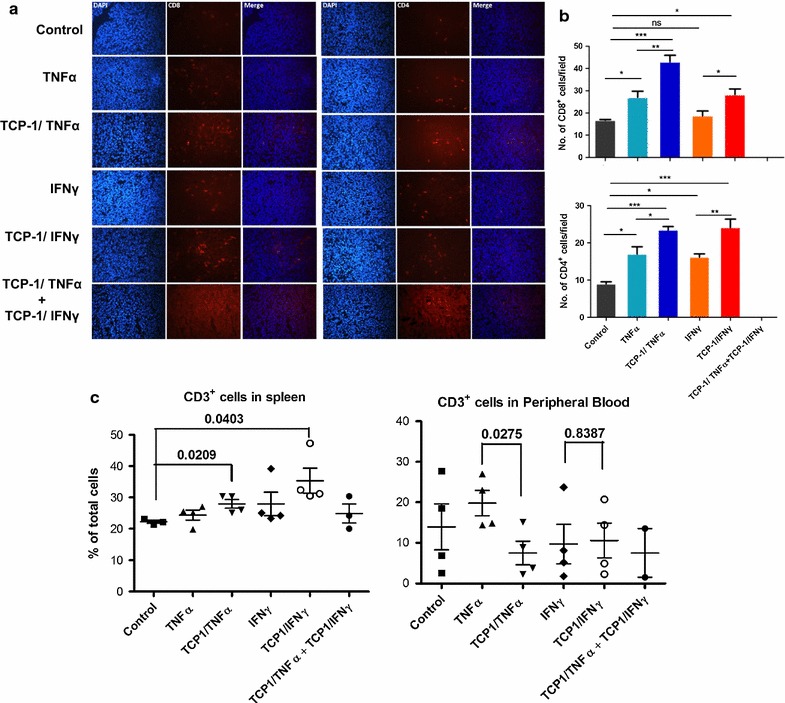
TNFα, TCP-1/TNFα, IFNγ or TCP-1/IFNγ single treatment enhanced antitumor immunity. **a** Immunofluorescence staining of CD8^+^ and CD4^+^ cells in the tumor mass. **b** Quantification of CD8^+^ and CD4^+^ cells in the tumor mass. TCP-1/TNFα and TCP-1/IFNγ single treatment significantly increased CD8^+^ and CD4^+^ cell infiltration into the tumor. **c** CD3^+^ cells in the spleen and peripheral blood detected by flow cytometry. TCP-1/TNFα and TCP-1/IFNγ single treatment increased CD3^+^ cells production in the spleen while decreased or didn’t change its level in peripheral blood, suggesting the treatments increased CD3^+^ cells at tumor site. Data were presented as mean ± SEM. *P < 0.05. **P < 0.01. ***P < 0.001

To investigate the immunotherapeutic responses, immune cell infiltration in the tumor was determined. Immunofluorescence staining showed that TNFα increased both CD8^+^ cytotoxic and CD4^+^ helper T lymphocytes inside the tumor while IFNγ increased CD4^+^ T cell. Compared with the unconjugated TNFα and IFNγ, the conjugated groups through TCP-1 further increased the infiltration of both CD8^+^ and CD4^+^ T cell in the tumor (Fig. 4a, b). However, there was no T lymphocyte infiltration observed in the TCP-1/TNFα and TCP-1/IFNγ combined group, suggesting the combined treatment group may not exert its antitumor effect through enhancing immune surveillance. The above result was further confirmed by flow cytometry (Fig. 4c). Both TCP-1/TNFα and TCP-1/IFNγ increased CD3^+^ cells in the spleen meaning more T lymphocytes were generated by the spleen. On the other hand, the peripheral blood cell analysis disclosed that there were more CD3^+^ cells in the peripheral blood for the TNFα treated group, indicating that fewer T lymphocytes has entered the tumor for this group. All other groups showed similar population of CD3^+^ cells in the peripheral blood compared with the control group. We have also determined the number of macrophages, NK cells, B cells and granulocytes inside the tumor, however, no obvious change was observed for all treatment groups (Additional file 5: Fig. S5). Taken together, the data suggest that TCP-1/TNFα and TCP-1/IFNγ could increase the generation of T lymphocyte in the spleen and enhance the penetration of T lymphocytes into the tumor. The TCP-1/TNFα and TCP-1/IFNγ combined treatment group inhibited tumor growth by different mechanisms from targeted TNFα or IFNγ alone which is highly associated with antitumor immunity.

### Mechanism of action of TCP-1/TNFα combined with TCP-1/IFNγ

**Fig. 5:**
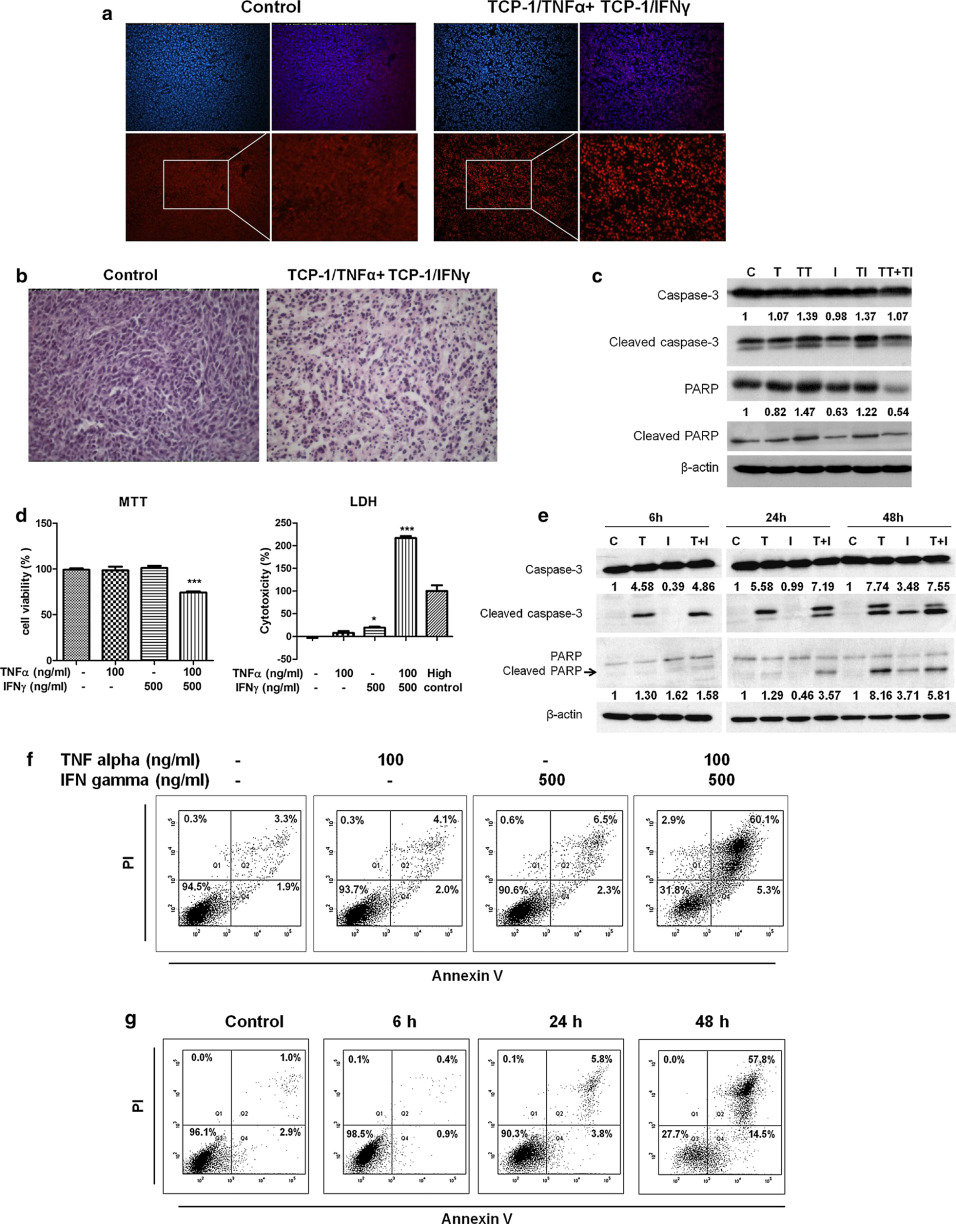
TCP-1/TNFα combined with TCP-1/IFNγ induced late apoptosis/secondary necrosis. **a** Hochest and PI double staining of tumor frozen sections from control and TCP-1/TNFα and TCP-1/IFNγ combined treatment groups. PI is only permeant to dead cells. PI positive staining was found in tumor cells in the TCP-1/TNFα and TCP-1/IFNγ combination treatment group but not control group indicating combination treatment induced late apoptosis/secondary necrosis. **b** H & E staining of tumor frozen sections. The combination treatment group had condensed (pyknosis) and fragmented nucleus (karyorrhexis) suggesting the cells were undergoing late apoptosis or necrosis. **c** Western blot result of cleaved caspase-3 and cleaved PARP in the tumors. Semi-quantification of cleaved caspase-3 and PARP is done using ImageJ software. TCP-1/TNFα and TCP-1/IFNγ single treatment induced apoptosis as shown by induction of cleaved caspase-3 and PARP while the combined treatment group did not show such effect. **d** MTT and LDH assay of colon 26 cells treated by PBS, TNFα, IFNγ or TNFα plus IFNγ. Ratio of TNFα to IFNγ was determined according to in vivo study. TNFα and IFNγ synergistically inhibited colon 26 cell growth and caused membrane damage at 48 h. **e** Western blot result of cleaved caspase-3 and cleaved PARP in colon 26 cells treated for different time. Semi-quantification of cleaved caspase-3 and PARP is done using ImageJ software. TNFα plus IFNγ induced apoptosis earlier than TNFα or IFNγ alone. **f** Flow cytometry result of Annexin V and PI double staining. TNFα plus IFNγ induced massive late apoptosis/necrosis at 48 h. **g** Time course study was performed with TNFα plus IFNγ at different time points. Results demonstrated that this cell death was through early apoptosis to late apoptosis/necrosis. *C* control, *T* TNFα, *I* IFNγ, *TT* TCP-1/TNFα, *TI* TCP-1/IFNγ. Data were presented as mean ± SEM. *P < 0.05. ***P < 0.001

To elucidate the mechanism by which TCP-1/TNFα combined with TCP-1/IFNγ induced massive cell death inside the tumor, Hochest and PI double stain was first performed in frozen tissue sections. PI is only permeant to dead cells. As shown in Fig. 5a, cells in the control tumor were not permeable to PI while cells in the combined treatment group were permeable to PI and had condensed (pyknosis) and fragmented nucleus (karyorrhexis) suggesting the cells were undergoing late apoptosis or necrosis. The morphology of the nucleaus was more clearly shown by H & E staining (Fig. 5b). Next, expression of apoptosis markers cleaved caspase-3 and PARP in the tumor were determined. Results showed that TCP-1/TNFα or TCP-1/IFNγ increased cleavage of caspase-3 and PARP while such effect was not found in the TCP-1/TNFα and TCP-1/IFNγ combined treatment group (Fig. 5c). On the contrary, there was a little reduction in the expression of total caspase-3, total PARP and cleaved PARP proteins. We hypothesized that the cells were at late stage of cell death and that the proteins in the cells were being degraded. To further clarify how the cells in the tumor died after TCP-1/TNFα and TCP-1/IFNγ combined treatment, we used the colon 26 cells to determine the effect of TNFα combined with IFNγ in vitro. We used the same ratio of TNFα to IFNγ as that of in vivo study (1:5). TNFα (100 ng/mL) and IFNγ (500 ng/mL) synergistically inhibited cell growth as shown by MTT assay (Fig. 5d). LDH assay is to detect plasma membrane damage which is a hallmark of late apoptosis/necrosis in vitro. Result of this assay showed that TNFα plus IFNγ induced plasma membrane damage to a great extent (Fig. 5d). Western blot result showed that combined treatment by TNFα and IFNγ induced caspase-3 and PARP cleavage which was earlier than either cytokine alone (Fig. 5e). Using flow cytometry of Annexn V and PI, we found that combined treatment by TNFα and IFNγ induced drastic cell death (late apoptosis or necrosis) at 48 h while the effect of both cytokines alone was minimal (Fig. 5f). Time course study showed that the cells went through early apoptosis to cell death (Fig. 5g). Moreover, the possibility of other cell death pathways including autophagy and senescence were excluded (Additional file 6: Fig. S6). Collectively, we conclude that the cells died by going through apoptosis not primary necrosis.

## Discussion

Tumor vasculature undergoing angiogenesis expresses specific endothelial surface markers which are absent or barely detectable in mature vessels [33]. Peptide has many advantages over antibody as drug carrier [34]. Therefore, tumor-homing peptides (THPs) that target tumor vasculature are important and promising imaging agent and drug delivery vectors [35]. Using phage display biopanning, we previously identified a novel cyclic peptide TCP-1 which can specifically bind to the vasculature of colorectal tumor in both animals and humans but not normal blood vessels. We have also shown that TCP-1 is useful for targeted delivery of imaging agent and pro-apoptotic peptide [17]. This peptide is advantageous over other THPs because it exhibits a unique homing ability to the vasculature only in the CRC, indicating its specificity and accuracy as a carrier in CRC diagnosis and therapy. The TNFα and IFNγ synergism has been reported under many biological conditions including their tumor inhibitory effect [25, 26, 36–38]. Early clinical trials conducted in 1990s tried to use the combination of these two cytokines to treat advanced gastrointestinal cancer patients [27–30]. Unfortunately only modest beneficial effects but severe side effects were seen. In vivo study showed that combination of nontargeted TNFα and IFNγ often show significant toxicity [39]. Targeted TNFα and nontargeted IFNγ exerted additive tumoricial activity in renal cell carcinoma [25]. However, direct proof of the antitumor effect of targeted TNFα combined with targeted IFNγ in vivo has not been reported before.

In this study, we extended to study the effect of TCP-1/TNFα and TCP-1/IFNγ either alone or in combination in orthotopic colorectal tumor model in immune-competent mice. We found that TNFα (1 μg/mouse) or IFNγ (5 μg/mouse) slightly increased tumor apoptosis while conjugation with TCP-1 peptide significantly inhibited tumor growth and increased tumor cell apoptosis. TNFα exerts different actions in cancer therapy depending on the dosage used [40]. High dose of TNFα mainly inhibits tumor angiogenesis, leading to vessel destruction which decreases the blood flow and oxygen required for the progression of tumor growth. Low dose of TNFα could induce vessel remodeling and increase vessel perfusion, thereby enhancing drug accumulation in the tumor. Our previous study has demonstrated that low dose TCP-1/TNFα could normalize tumor blood vessel, enhance the intratumoral accumulation of anticancer drug 5-FU, thus potentiating its antitumor activity [18]. In the present study, there was no significantly vessel destruction observed for TNFα or TCP-1/TNFα. Our previous result demonstrated that TNFα or TCP-1/TNFα at 5 μg/mouse could lead to acute vessel destruction. We here chose 1 μg/mouse for TNFα which could partly inhibit tumor growth and avoid masking the action of combined treatment of TCP-1/TNFα and TCP-1/IFNγ. This dosage may not be sufficient to induce vessel destruction. For IFNγ, consistent with previous findings that a dose–response curve of IFNγ-NGR is bell-shaped [23], our result also showed that TCP-1/IFNγ achieved optimal antitumor effect at the dose of 5 μg/mouse. Elevating the dose did not ensure better response. In addition to inducing tumor apoptosis, TCP-1/TNFα or TCP-1/IFNγ also increased the infiltration of CD8^+^ and CD4^+^ T cell in the tumor leading to enhanced antitumor immunity. Previous study has shown that the antitumor effect of targeted IFNγ in animal study mainly involves inhibition of angiogenesis and induction of apoptosis but not infiltration of immune cells [22, 40], which is different from our result. This difference may be due to the different dose, treatment time and animal model.

Most interestingly, combination treatment by TCP-1/TNFα and TCP-1/IFNγ dramatically inhibited tumor growth. At the beginning we used TCP-1/TNFα (1 μg/mouse) combined with TCP-1/IFNγ (5 μg/mouse). The antitumor effect was very drastic, however a little myelosuppression was observed (data not shown). After that, we used half the dose: TCP-1/TNFα (0.5 μg/mouse) combined with TCP-1/IFNγ (2.5 μg/mouse). The inhibitory effect of combination treatment on tumor growth was dose-dependent (data not shown) and no myelosuppression or toxicity to other organs was observed for the latter combination. TCP-1/TNFα and TCP-1/IFNγ combined treatment also significantly improved maximum tolerance of two cytokines related to side effects compared with untargeted TNFα combined with IFNγ indicating alleviation of systematic toxicity. Morphologically, condensed and fragmented nucleus (pyknosis and karyorrhexis) were found in high percentage (>90 %) inside the tumor treated by combined treatment which might be due to late apoptosis or necrosis. Mechanism study using mouse colon cancer cells, colon 26 cells reveals that the cells treated with TNFα and IFNγ pass through early apoptosis to late apoptosis/secondary necrosis but not through primary necrosis.

Although we have shown that combined treatment of TCP-1/TNFα and TCP-1/IFNγ induced late apoptosis/secondary necrosis, the molecular mechanism remains elusive. According to previous studies, TNFα induced NF-κB signaling could counteract TNFα-induced apoptosis [41, 42] while IFNγ could inhibit NF-κB activation and the expression of downstream apoptosis inhibitors, finally sensitizing the cancer cells to TNFα treatment [24, 26]. However, our results showed that the expression of apoptosis inhibitor downstream of NF-κB including XIAP and c-IAP-1 were not changed by either TNFα or combined treatment (Additional file 6: Fig. S6). It has also been proposed that TNFα and IFNγ synergistically inhibit cancer cell growth because IFNγ increases the expression of TNFα receptors [43, 44]. However, it is also argued that increase of TNFα receptors is not the major mechanism underlying the synergism between TNFα and IFNγ. STAT1/IRF-1 pathways initiated by IFNγ had been shown to be important in TNFα and IFNγ synergism in inducing cervical cancer cell apoptosis [26]. However, neither STAT1 nor IRF-1 could totally explain the priming effect of IFNγ in TNFα-indiced apoptosis. It seems that the TNFα and IFNγ synergism requires multiple factors, and research evidence so far has not pointed to a conclusion. Further studies are warranted to delineate the potential molecular mechanism.

## Conclusions

Our results demonstrate for the first time that TCP-1/TNFα and TCP-1/IFNγ combination is very promising as potential CRC therapy. The synergistic antitumor activity of TNFα combined with IFNγ has been exploited in early clinical trials, but the result was disappointing due to systematic toxicity and limited beneficial effect. Our study could overcome this drawback by enhancing the anticancer action of TNFα combining with IFNγ so that lower doses of both cytokines can be given to patients to achieve promising outcome with less systemic side effects. Since both cytokines are being used in human to treat different diseases, the approach suggested from the current study would provide the likelihood of using drug combination of TCP-1/TNFα and TCP-1/IFNγ for future clinical trials to treat CRC.

## Additional files

10.1186/s12967-016-0944-3 Schematic illustration of plasmid construction. TNFα or IFNγ was amplified by PCR with respective primers containing different restriction enzyme sequences as shown in the figure. TNFα or IFNγ was then cloned into a modified pET-14b vector after restriction digestion. The TCP-1 gene was introduced into the constructed TNFα or IFNγ-pET-14b plasmid through PCR-based site-directed mutagenesis.
10.1186/s12967-016-0944-3 IFNγ or TCP-1/IFNγ did not bind to control organs including brain, colon and heart. IFNγ or TCP-1/IFNγ was detected by anti-His tag antibody (green). Staining of nuclei was performed with DAPI.
10.1186/s12967-016-0944-3 Antitumor activity of IFNγ and TCP-1/IFNγ at different dose in the orthotopic CRC model. (A) Picture of the tumors after 7 day treatment (n ≥ 2 per group). (B) Relative tumor mass at the end of experiment. TCP-1/IFNγ at 5 μg/mouse significantly decreased tumor volume. *P < 0.05.
10.1186/s12967-016-0944-3 Antitumor activity of TCP-1/TNFα combined with TCP-1/IFNγ compared with single treatment and histological examination of combined treatment. (A) Picture of the tumors after 7 day treatment (n ≥ 3 per group). (B) Tumor weight at the end of experiment. Combined treatment (TCP-1/TNFα 1 μg/mouse and TCP-1/IFNγ 5 μg/mouse) significantly inhibited tumor growth than single treatment. (C) Body weight change of TCP-1/TNFα and TCP-1/IFNγ combined treatment group (TCP-1/TNFα 0.5 μg/mouse and TCP-1/IFNγ 2.5 μg/mouse) compared with control group. (D) Sections from TCP-1/TNFα and TCP-1/IFNγ combined treatment group (TCP-1/TNFα 0.5 μg/mouse and TCP-1/IFNγ 2.5 μg/mouse) were subsequently stained by Harris hematoxylin solution and eosin Y solution (H & E). There were no detectable pathological changes in the control and treatment group.
10.1186/s12967-016-0944-3 TCP-1/TNFα or TCP-1/IFNγ did not induce infiltration of macrophages, NK cells, granulocytes or B cells into the tumor. (A) Flow cytometry of CD68^+^ cell showing the number of macrophage in the spleen and tumor. (B) Immunohistochemistry staining of CD335^+^ NK cells in the tumor. (C) Immunofluorescence staining of Ly6G and CD19 showing the granulocytes and B cells in the tumor respectively.
10.1186/s12967-016-0944-3 Western blot result of autophagy and senescence markers and apoptosis inhibitors. (A) TCP-1/TNFα and TCP-1/IFNγ alone or in combination did not affect the expression of autophagy markers including LC3B and p62 and senescence markers including p53, cyclin E or CDK2 in the tumor. (B) Western blot result of autophagy and senescence markers in colon 26 cells treated with TNFα and IFNγ alone or in combination at 6 and 24 h post treatment. No effect on the autophagy and senescence markers or apoptosis inhibitor including XIAP and c-IAP-1 was found. C: control, T: TNFα, I: IFNγ, TT: TCP-1/TNFα, TI: TCP-1/IFNγ.

## Abbreviations

TNFα: tumor necrosis factor alpha
IFNγ: interferon gamma
CRC: colorectal cancer
ILP: isolated limb perfusion
5-FU: 5-fluorouracil
NF-κB: nuclear factor kappa-light-chain-enhancer of activated B cells
XIAP: X-linked inhibitor of apoptosis protein
c-IAP-1: cellular inhibitor of apoptosis protein-1
STAT1: signal transducer and activator of transcription 1
IRF-1: interferon regulatory factor 1
