# Supplementary material for: Vascular-targeted TNFα and IFNγ inhibits orthotopic colorectal tumor growth
Source: J Transl Med. 2016 Jun 24;14:187. doi: 10.1186/s12967-016-0944-3 (PMC4919862; doi:10.1186/s12967-016-0944-3)
Supplement: Supplementary file 1 — 10.1186/s12967-016-0944-3 Schematic illustration of plasmid construction. TNFα or IFNγ was amplified by PCR with respective primers containing different restriction enzyme sequences as shown in the figure. TNFα or IFNγ was then cloned into a modified pET-14b vector after restriction digestion. The TCP-1 gene was introduced into the constructed TNFα or IFNγ-pET-14b plasmid through PCR-based site-directed mutagenesis. [file 12967_2016_944_MOESM1_ESM.pptx]

## Slide 1
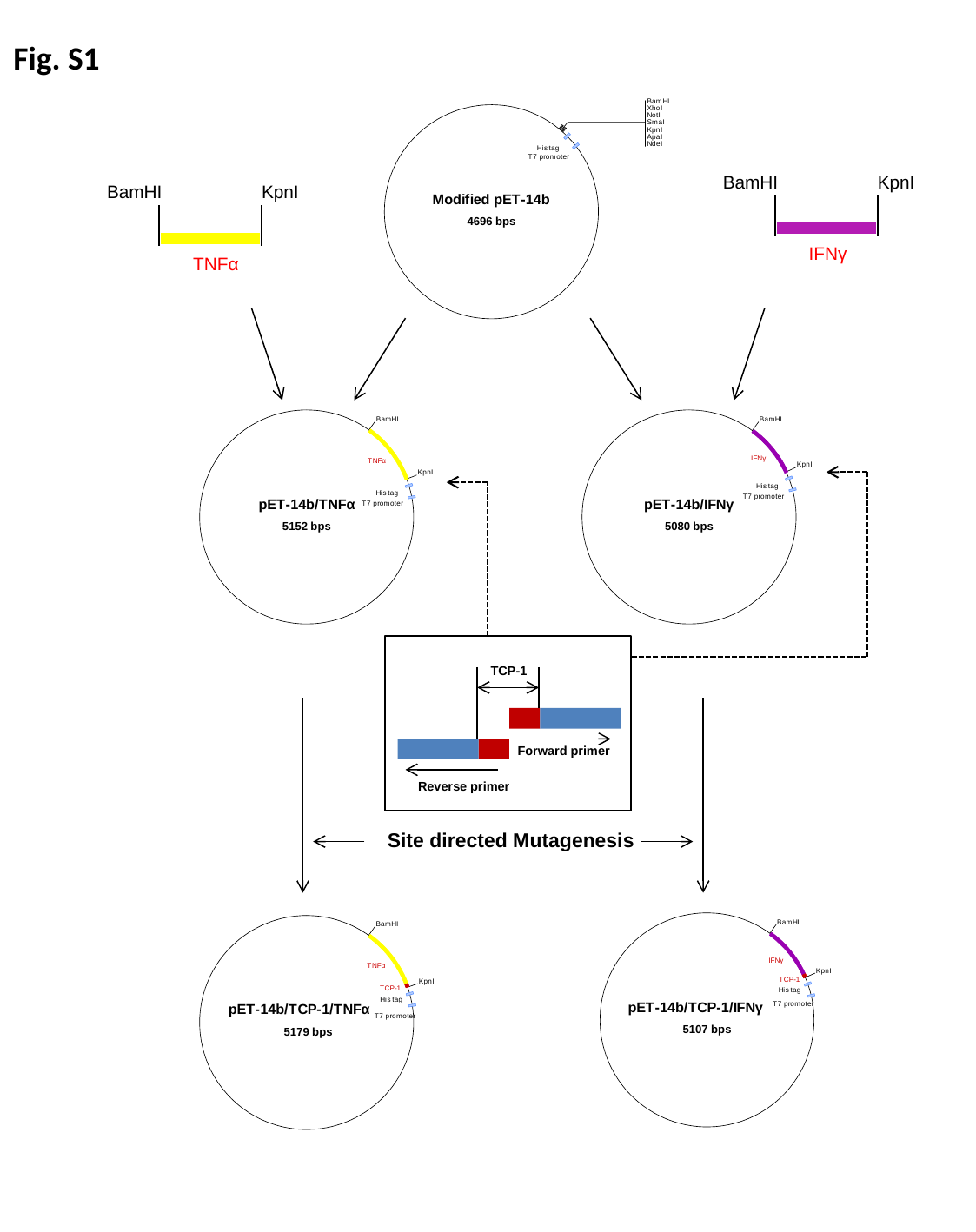

Fig. S1
BamHI KpnI
BamHI KpnI
IFNγ
TNFα
TCP-1
Forward primer
Reverse primer
Site directed Mutagenesis
