# Supplementary figures and images for: Vascular-targeted TNFα and IFNγ inhibits orthotopic colorectal tumor growth
Source: J Transl Med. 2016 Jun 24;14:187. doi: 10.1186/s12967-016-0944-3 (PMC4919862; doi:10.1186/s12967-016-0944-3)

## Slide 1
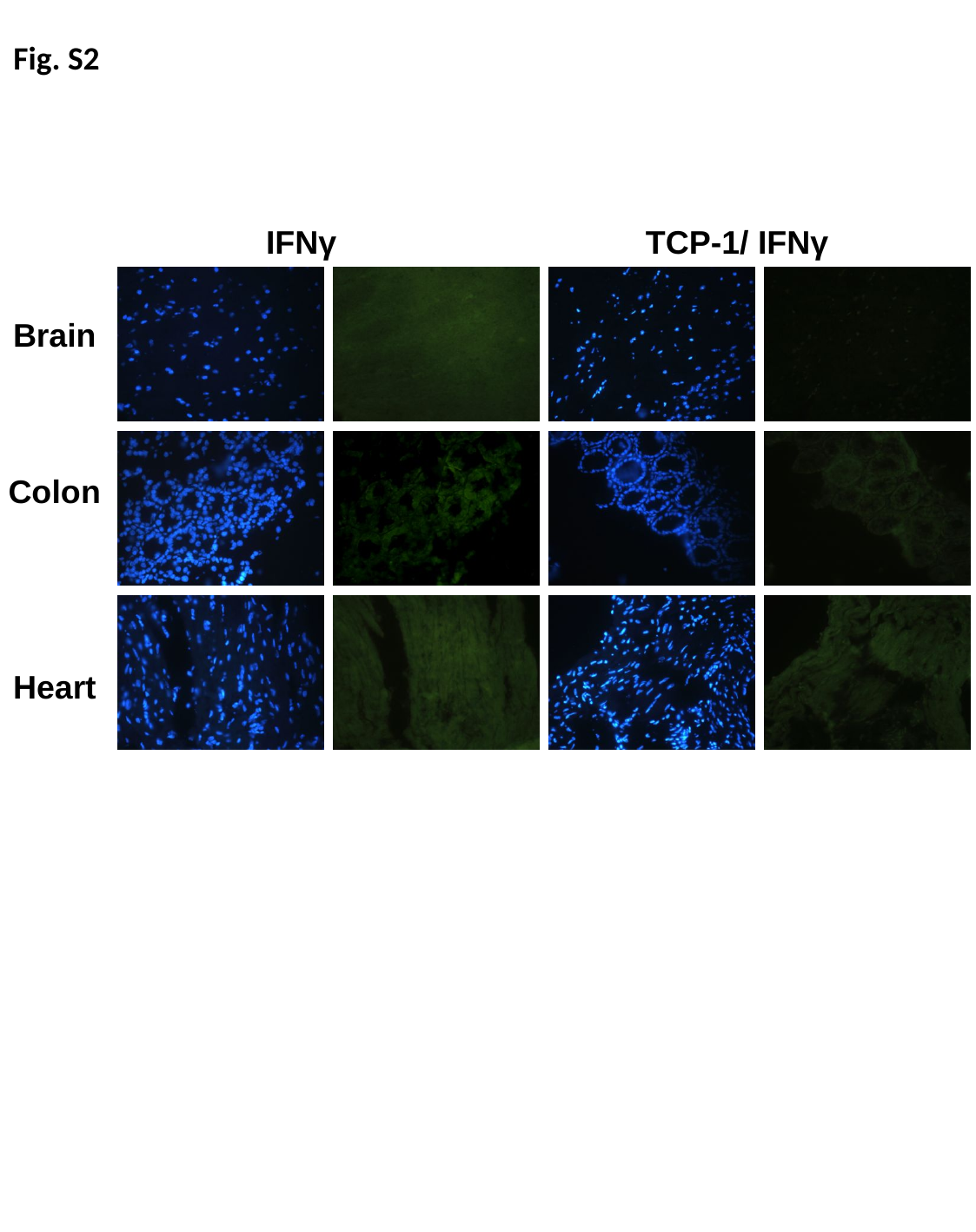

Fig. S2
IFNγ TCP-1/ IFNγ
Brain
Colon
Heart

Supplement: Supplementary file 2 — 10.1186/s12967-016-0944-3 IFNγ or TCP-1/IFNγ did not bind to control organs including brain, colon and heart. IFNγ or TCP-1/IFNγ was detected by anti-His tag antibody (green). Staining of nuclei was performed with DAPI. [file 12967_2016_944_MOESM2_ESM.pptx]

## Slide 1
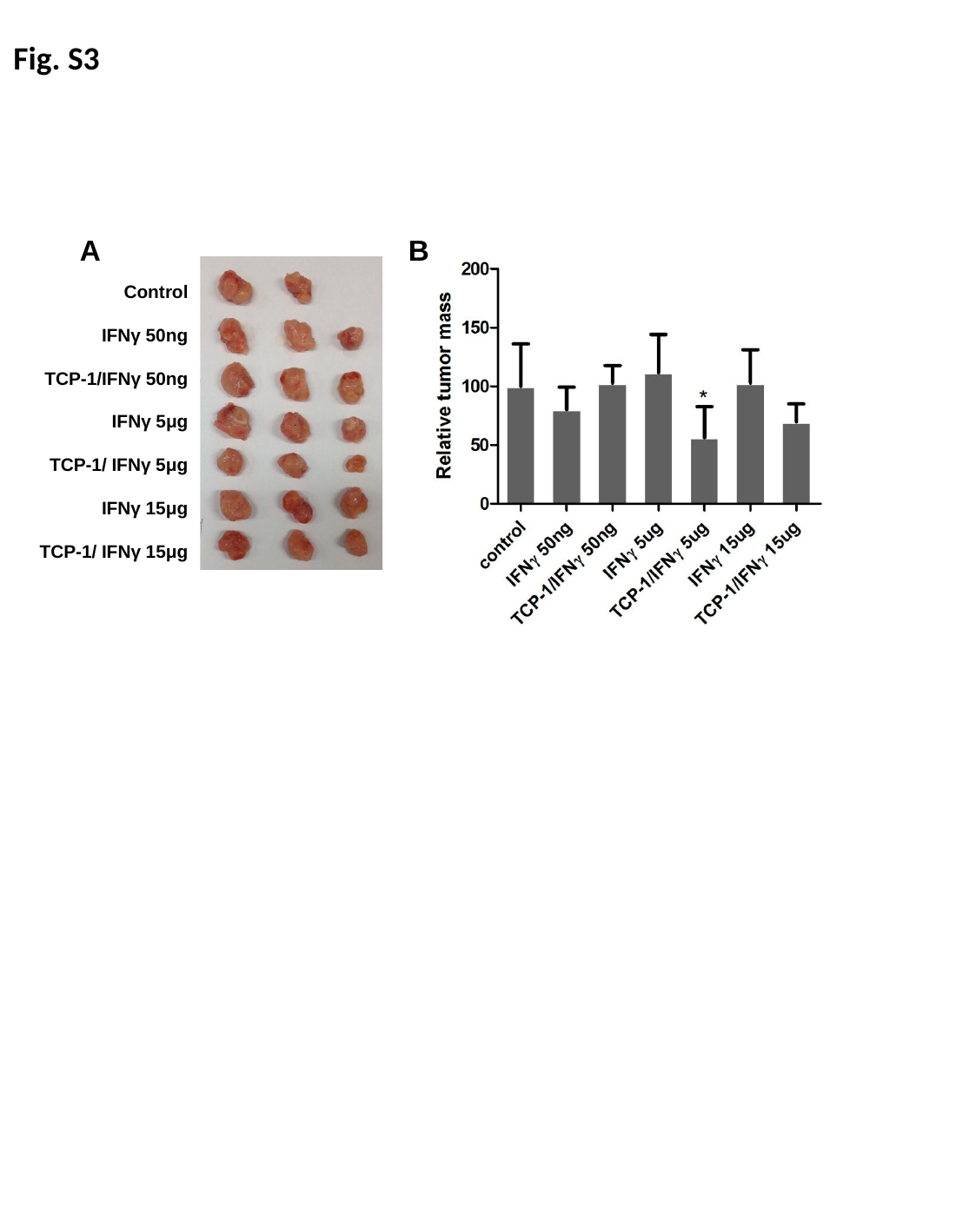

Fig. S3
A
B
Control
IFNγ 50ng
TCP-1/IFNγ 50ng
IFNγ 5μg
TCP-1/ IFNγ 5μg
IFNγ 15μg
TCP-1/ IFNγ 15μg

Supplement: Supplementary file 3 — 10.1186/s12967-016-0944-3 Antitumor activity of IFNγ and TCP-1/IFNγ at different dose in the orthotopic CRC model. (A) Picture of the tumors after 7 day treatment (n ≥ 2 per group). (B) Relative tumor mass at the end of experiment. TCP-1/IFNγ at 5 μg/mouse significantly decreased tumor volume. *P < 0.05. [file 12967_2016_944_MOESM3_ESM.pptx]

## Slide 1
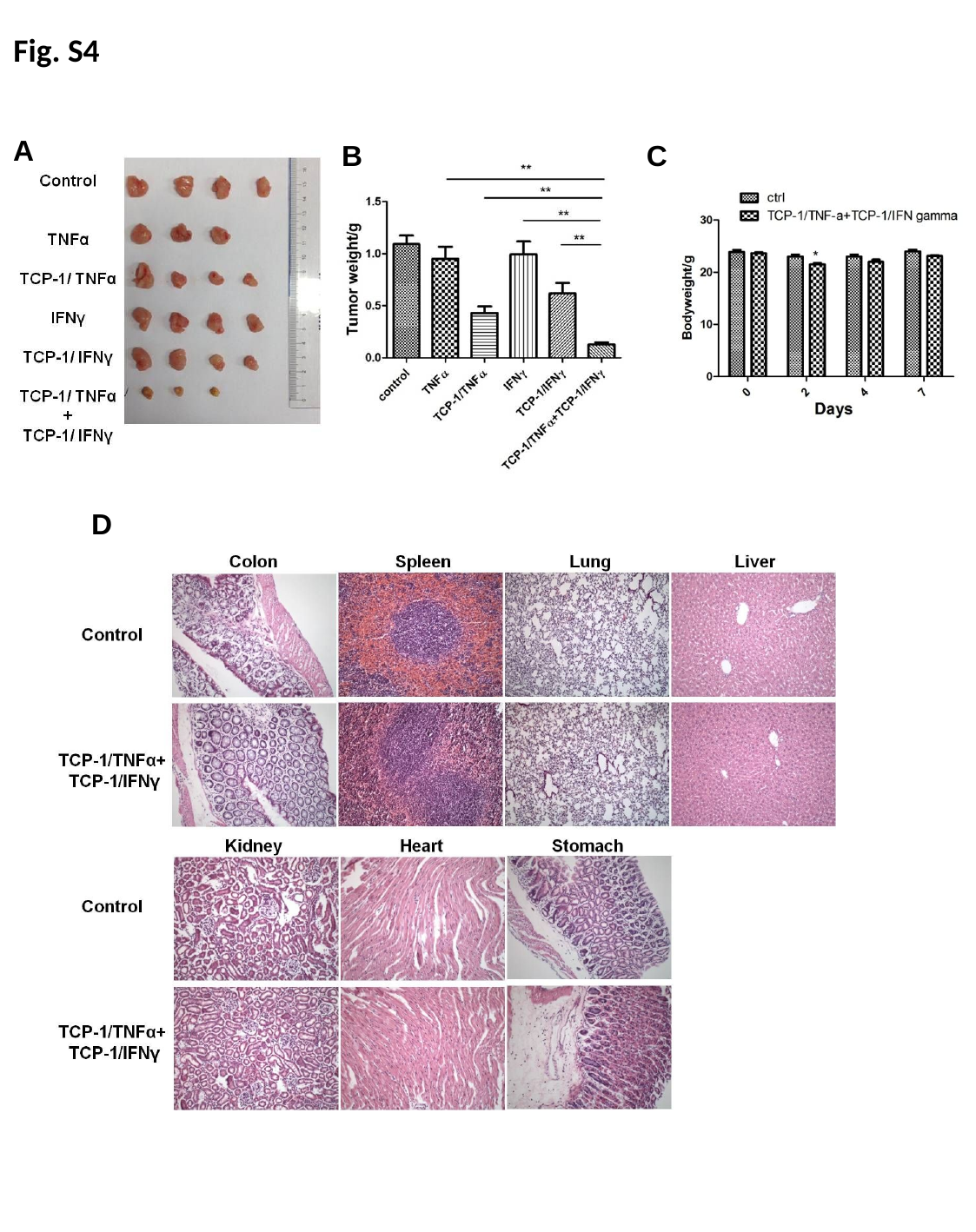

Fig. S4
A
B
C
D

Supplement: Supplementary file 4 — 10.1186/s12967-016-0944-3 Antitumor activity of TCP-1/TNFα combined with TCP-1/IFNγ compared with single treatment and histological examination of combined treatment. (A) Picture of the tumors after 7 day treatment (n ≥ 3 per group). (B) Tumor weight at the end of experiment. Combined treatment (TCP-1/TNFα 1 μg/mouse and TCP-1/IFNγ 5 μg/mouse) significantly inhibited tumor growth than single treatment. (C) Body weight change of TCP-1/TNFα and TCP-1/IFNγ combined treatment group (TCP-1/TNFα 0.5 μg/mouse and TCP-1/IFNγ 2.5 μg/mouse) compared with control group. (D) Sections from TCP-1/TNFα and TCP-1/IFNγ combined treatment group (TCP-1/TNFα 0.5 μg/mouse and TCP-1/IFNγ 2.5 μg/mouse) were subsequently stained by Harris hematoxylin solution and eosin Y solution (H & E). There were no detectable pathological changes in the control and treatment group. [file 12967_2016_944_MOESM4_ESM.pptx]

## Slide 1
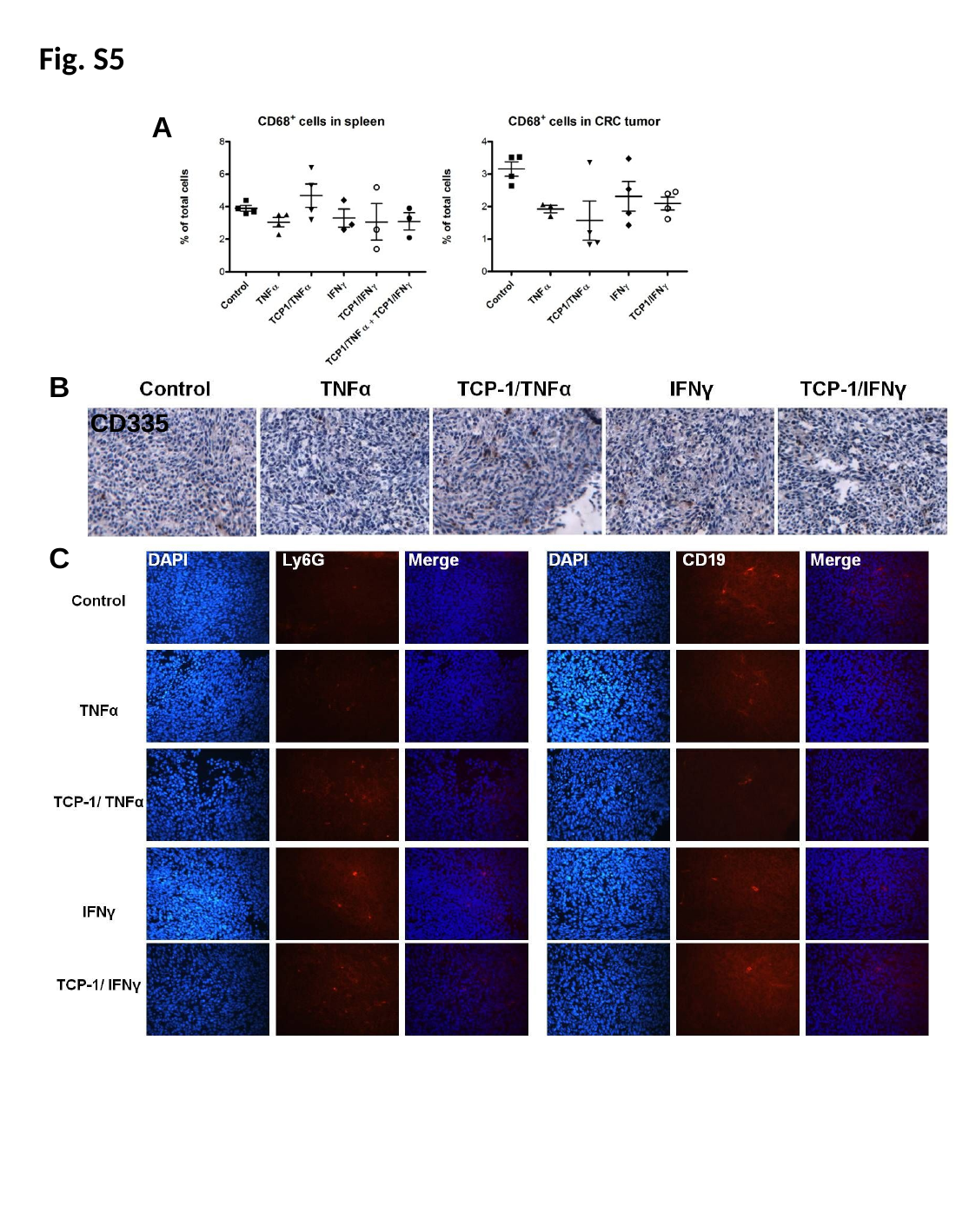

Fig. S5
A
B
CD335
C

Supplement: Supplementary file 5 — 10.1186/s12967-016-0944-3 TCP-1/TNFα or TCP-1/IFNγ did not induce infiltration of macrophages, NK cells, granulocytes or B cells into the tumor. (A) Flow cytometry of CD68+ cell showing the number of macrophage in the spleen and tumor. (B) Immunohistochemistry staining of CD335+ NK cells in the tumor. (C) Immunofluorescence staining of Ly6G and CD19 showing the granulocytes and B cells in the tumor respectively. [file 12967_2016_944_MOESM5_ESM.pptx]

## Slide 1
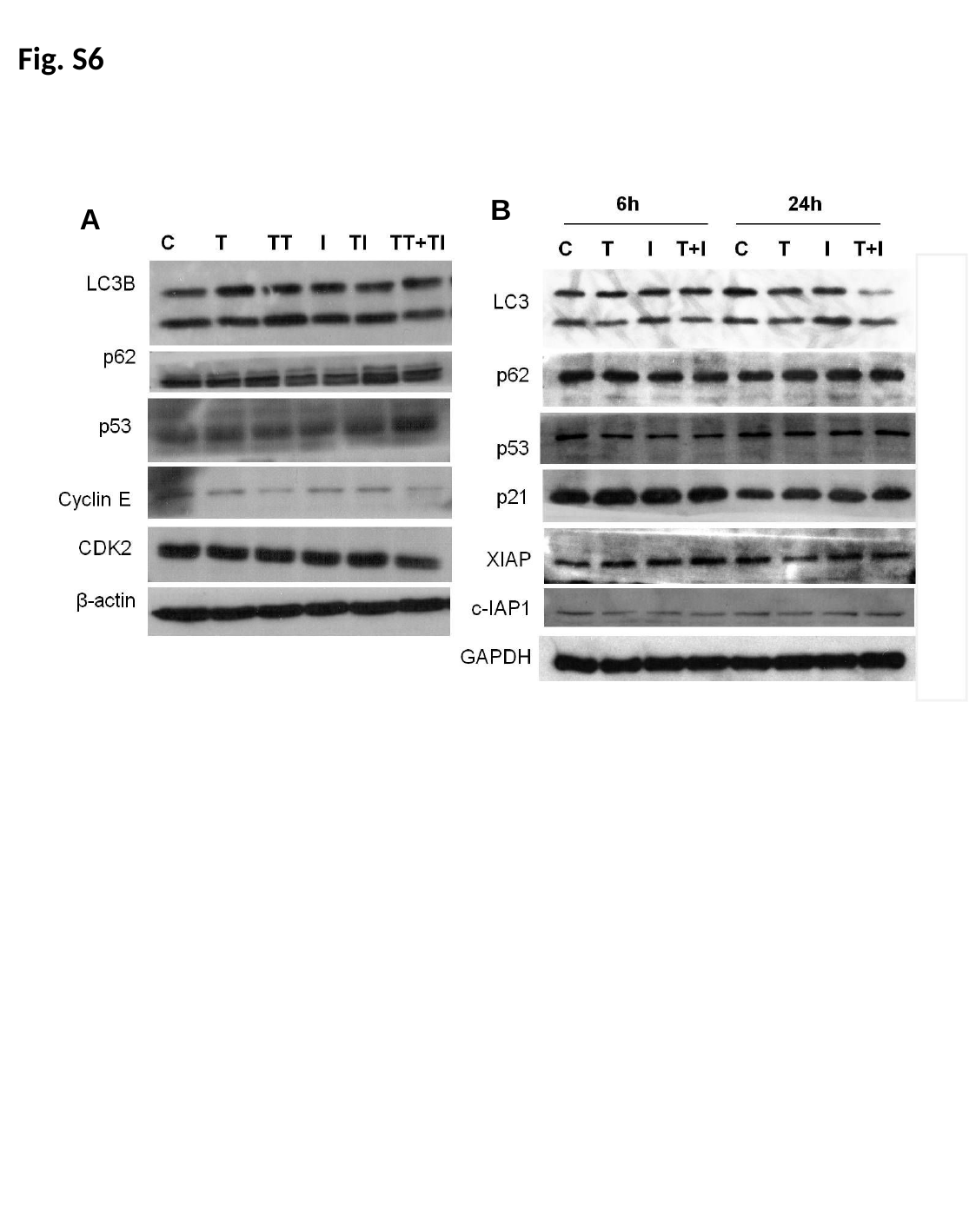

Fig. S6
B
A

Supplement: Supplementary file 6 — 10.1186/s12967-016-0944-3 Western blot result of autophagy and senescence markers and apoptosis inhibitors. (A) TCP-1/TNFα and TCP-1/IFNγ alone or in combination did not affect the expression of autophagy markers including LC3B and p62 and senescence markers including p53, cyclin E or CDK2 in the tumor. (B) Western blot result of autophagy and senescence markers in colon 26 cells treated with TNFα and IFNγ alone or in combination at 6 and 24 h post treatment. No effect on the autophagy and senescence markers or apoptosis inhibitor including XIAP and c-IAP-1 was found. C: control, T: TNFα, I: IFNγ, TT: TCP-1/TNFα, TI: TCP-1/IFNγ. [file 12967_2016_944_MOESM6_ESM.pptx]
